# National standardisierter Broad Consent in der Praxis: erste Erfahrungen, aktuelle Entwicklungen und kritische Betrachtungen

**DOI:** 10.1007/s00103-024-03878-6

**Published:** 2024-04-19

**Authors:** Sven Zenker, Daniel Strech, Roland Jahns, Gabriele Müller, Fabian Prasser, Christoph Schickhardt, Georg Schmidt, Sebastian C. Semler, Eva Winkler, Johannes Drepper

**Affiliations:** 1 Stabsstelle Medizinisch-Wissenschaftliche Technologieentwicklung und -koordination (MWTek), Kaufmännische Direktion, Universitätsklinikum Bonn, Bonn, Deutschland; 2 AG Angewandte Medizininformatik (AMI), Institut für Medizinische Biometrie, Informatik und Epidemiologie (IMBIE), Rheinische Friedrich-Wilhelms-Universität Bonn, Bonn, Deutschland; 3 AG Angewandte Mathematische Physiologie (AMP), Klinik & Poliklinik für Anästhesiologie und Operative Intensivmedizin, Universitätsklinikum Bonn, Bonn, Deutschland; 4 QUEST Center for Responsible Research, Berlin Institute of Health at Charité – Universitätsmedizin Berlin, Berlin, Deutschland; 5 Interdisziplinäre Biomaterial- und Datenbank Würzburg (ibdw), Universitätsklinikum Würzburg, Würzburg, Deutschland; 6 Zentrum für Evidenzbasierte Gesundheitsversorgung, Universitätsklinikum und Medizinische Fakultät Carl Gustav Carus an der Technischen Universität Dresden, Dresden, Deutschland; 7 Center for Health Data Science, Berlin Institute of Health at Charité – Universitätsmedizin Berlin, Berlin, Deutschland; 8 Sektion Translationale Medizinethik, KKE Angewandte Tumor-Immunität, Nationales Centrum für Tumorerkrankungen (NCT), Deutsches Krebsforschungszentrum (DKFZ) Heidelberg und Universitätsklinikum Heidelberg, Heidelberg, Deutschland; 9 Klinik und Poliklinik für Innere Medizin I. Klinikum rechts der Isar der Technischen Universität München, München, Deutschland; 10 TMF – Technologie- und Methodenplattform für die vernetzte medizinische Forschung e. V., Berlin, Deutschland; 11 Sektion Translationale Medizinethik, Abteilung Medizinische Onkologie, Nationales Centrum für Tumorerkrankungen (NCT), Universitätsklinikum Heidelberg und Universität Heidelberg, Heidelberg, Deutschland; 12 Stabsstelle Medizinisch-Wissenschaftliche Technologieentwicklung und -koordination (MWTek) Kaufmännische Direktion, Universitätsklinikum Bonn, Venusberg-Campus 1, 53127 Bonn, Deutschland

**Keywords:** Informierte Einwilligung, Sekundärnutzung von Gesundheitsdaten, Breite Zweckbeschreibung, Datenschutz-Grundverordnung, Implementierungsbegleitung, Informed consent, Secondary use of health data, Broad description of purpose, General Data Protection Regulation, Implementation support

## Abstract

**Hintergrund:**

Die Digitalisierung im Gesundheitswesen verspricht eine Sekundärnutzung von Patientendaten im Sinne eines lernenden Gesundheitssystems. Die Arbeitsgruppe Consent der Medizininformatik-Initiative (MII) hat mit einheitlichen Einwilligungsdokumenten eine ethische und rechtliche Grundlage hierfür geschaffen. Beschrieben wird die systematisch begleitete Einführung dieser Dokumente an den Standorten der MII.

**Methoden:**

Die Begleitung der Einführung umfasste regelmäßige Online-Umfragen, die vertiefte Analyse der Einführungsprozesse an ausgewählten Standorten sowie die Untersuchung der jeweils eingesetzten Dokumente. Zudem wurden Anfragen und Rückmeldungen einer Vielzahl von Stakeholdern ausgewertet.

**Ergebnisse:**

Die Online-Umfragen ergaben, dass 27 der 32 befragten Standorte nach und nach die Einwilligungsdokumente produktiv eingeführt haben, mit aktuell insgesamt 173.289 Einwilligungen. Die Analyse der Umsetzungsverfahren offenbarte heterogene organisatorische Rahmenbedingungen an den Standorten. Anforderungen verschiedener Stakeholder konnten durch die Erarbeitung und Bereitstellung ergänzender Versionen der Einwilligungsdokumente und zusätzlicher Informationsmaterialien erfüllt werden.

**Diskussion:**

Die Einführung der Einwilligungsdokumente der MII an den Universitätskliniken schafft eine einheitliche Rechtsgrundlage für die Sekundärnutzung von Patientendaten. Die flächendeckende Implementierung innerhalb der Standorte bleibt jedoch herausfordernd. Minimalanforderungen an die Patientenaufklärung und ergänzende Best-Practice-Empfehlungen sind hierfür zu erarbeiten. Die Weiterentwicklung des nationalen Rechtsrahmens für die Forschung wird die hier entwickelten Mitsprache- und Transparenzmechanismen für Betroffene nicht obsolet machen.

**Zusatzmaterial online:**

Zusätzliche Informationen sind in der Online-Version dieses Artikels (10.1007/s00103-024-03878-6) enthalten.

## Abstract (en)

**Background:**

The digitalization in the healthcare sector promises a secondary use of patient data in the sense of a learning healthcare system. For this, the Medical Informatics Initiative’s (MII) Consent Working Group has created an ethical and legal basis with standardized consent documents. This paper describes the systematically monitored introduction of these documents at the MII sites.

**Methods:**

The monitoring of the introduction included regular online surveys, an in-depth analysis of the introduction processes at selected sites, and an assessment of the documents in use. In addition, inquiries and feedback from a large number of stakeholders were evaluated.

**Results:**

The online surveys showed that 27 of the 32 sites have gradually introduced the consent documents productively, with a current total of 173,289 consents. The analysis of the implementation procedures revealed heterogeneous organizational conditions at the sites. The requirements of various stakeholders were met by developing and providing supplementary versions of the consent documents and additional information materials.

**Discussion:**

The introduction of the MII consent documents at the university hospitals creates a uniform legal basis for the secondary use of patient data. However, the comprehensive implementation within the sites remains challenging. Therefore, minimum requirements for patient information and supplementary recommendations for best practice must be developed. The further development of the national legal framework for research will not render the participation and transparency mechanisms developed here obsolete.

## Hintergrund

Das vielleicht wichtigste Versprechen der Digitalisierung im Gesundheitssystem jenseits einer besseren Patientenversorgung ist die Gewinnung wissenschaftlicher Erkenntnisse durch die Sekundärnutzung von Patientendaten aus der Routineversorgung, also die Nutzung von Daten aus der Patientenversorgung für andere als den primären Erhebungszweck. Bislang stehen diese Daten für standortübergreifende Auswertungen aus rechtlichen, organisatorischen und technischen Gründen kaum zur Verfügung. Die Medizininformatik-Initiative (MII) hat sich daher als ein zentrales Ziel gesetzt, die breite Sekundärnutzung von Patientendaten für Forschungszwecke zu ermöglichen ([1]; siehe auch Beitrag von Semler et al. in diesem Themenheft).

Um eine solche Sekundärnutzung auf eine datenschutzrechtlich abgesicherte Grundlage zu stellen, wurde durch das Nationale Steuerungsgremium (NSG) der MII direkt zu Beginn der Förderinitiative die Arbeitsgruppe (AG) Consent ins Leben gerufen. Auch wenn der Name eine Vorfestlegung auf eine informierte Einwilligung als Rechtsgrundlage vermuten lässt, hat die AG doch zuvor alternative Rechtsgrundlagen sorgfältig geprüft. Die seit 2018 zur Anwendung kommende und mit dem Ziel der europaweiten Harmonisierung datenschutzrechtlicher Rahmenbedingungen gestartete Datenschutz-Grundverordnung (DSGVO) der Europäischen Union (EU) schafft jenseits der Einwilligung keine direkt anwendbare Rechtsgrundlage für die Sekundärnutzung von Patientendaten zu Forschungszwecken. Auf Basis der Öffnungsklausel in Artikel 9 Abs. 2 Buchst. j DSGVO können die EU und die Mitgliedsstaaten allerdings unter bestimmten Voraussetzungen solche Rechtsgrundlagen für die Nutzung von Gesundheitsdaten unabhängig von einer Einwilligung schaffen. In Deutschland finden sich auf dieser Grundlage basierende Regelungen im Bundesdatenschutzgesetz (BDSG; [2]) sowie in den Landesdatenschutzgesetzen und auch im Landeskrankenhausrecht. Diese Forschungsklauseln sind allerdings im Detail sehr unterschiedlich formuliert. Eine bundeslandübergreifend einheitliche und verlässliche Nutzung ist damit praktisch ausgeschlossen [3, 4]. Zudem adressieren viele dieser Regelungen vorzugsweise die Eigenforschung im Krankenhaus. Eine Herausgabe von Patientendaten ohne Einwilligung für ein konkretes Forschungsprojekt ist nur in engen Grenzen und unter ganz bestimmten Voraussetzungen in ausgewählten Bundesländern möglich (z. B. [5]). Hinweise darauf, wie diese Regelungen ausgebaut und perspektivisch genutzt werden könnten [6–8], helfen bei der Implementierung einer Lösung in einem vorgegebenen Förderzeitraum naturgemäß nicht. Auch die Anonymisierung der Patientendaten stellt für die Sekundärnutzung keine zufriedenstellende Lösung dar, da damit in aller Regel aufwändige Prüfungen der Anonymität der zu nutzenden Daten in allen Einzelfällen nötig werden und zudem eine sorgfältige Anonymisierung der Daten häufig zum Verlust wissenschaftlich relevanter Information führt [9, 10].

Vor diesem Hintergrund hat die AG Consent auf der Grundlage vorhandener und bewährter Einwilligungstexte für die breite Sekundärnutzung von Patientendaten und Bioproben [11–14] modulare Einwilligungsdokumente, bestehend aus Patienteninformation und Einwilligungserklärung, erstellt und im Rahmen eines mehrjährigen, deutschlandweiten Dialogs insbesondere mit Ethikkommissionen und Datenschutzbehörden weiterentwickelt und abgestimmt. Die im Dokument aufgeführten Module zur Nutzung von Bioproben oder Krankenkassendaten können standortspezifisch ausgewählt oder weggelassen werden.

Neben den Einwilligungsdokumenten, die einen *Broad Consent* (breite Einwilligung) darstellen (siehe Infobox), ist darüber hinaus ein Rahmen begleitender Schutz- und Transparenzmaßnahmen entstanden, der den von den Datenschutzbehörden geforderten Kompensationsmaßnahmen beim Einsatz breiter Forschungseinwilligungen [15] entspricht und in einer Handreichung zum Einsatz der Einwilligungsdokumente verbindlich beschrieben wurde [16, 17]. Die Konferenz der unabhängigen Datenschutzbehörden des Bundes und der Länder (Datenschutzkonferenz, DSK) hat im April 2020 einstimmig festgestellt, dass gegen die Umsetzung dieses Konzepts und den Einsatz der Einwilligungsdokumente in der Version 1.6d samt Handreichung in der Version 0.9d keine Bedenken bestehen.^1^

Im Folgenden wird beschrieben, wie die Einführung des Einsatzes dieser Einwilligungsdokumente an den beteiligten Standorten in der MII von der AG Consent begleitet wurde und wie daraus gewonnene Erfahrungen für die Weiterentwicklung der Texte, begleitender Materialien sowie der Rahmenbedingungen nutzbar gemacht wurden.

## Methoden

### Beobachtung und Analyse der Einführung

Die AG Consent, die sich interdisziplinär aus den in der MII organisierten Projekten und Konsortien zusammensetzt und sich in der Regel 5‑mal im Jahr virtuell oder physisch trifft, steht im Zentrum der laufenden Begleitung und Untersuchung der Einführung der Dokumente an den Standorten. Die Koordinierung der Arbeitsgruppe erfolgt durch die TMF – Technologie- und Methodenplattform für die vernetzte medizinische Forschung e. V.

#### Standortbefragungen

Ab August 2020 wurde vor den Sitzungen der AG Consent jeweils mit ca. 14-Tage-Frist eine Online-Umfrage zur Einführung der Dokumente an den Standorten durchgeführt. Verwendet wurde hierfür die Plattform SoSci Survey.^2^ Standortbezogene Links erlaubten die Beantwortung der Fragen durch unterschiedliche Personen am Standort und die Beibehaltung der Daten aus den Vorerhebungen, sodass lediglich noch Aktualisierungen einzelner Angaben vorzunehmen waren. Bis zum November 2023 wurden so 15 Erhebungen durchgeführt. Die Ergebnisse wurden jeweils in den Sitzungen der AG Consent vorgestellt und diskutiert. Auf dieser Basis sowie auf Basis weiterer Rückmeldungen erfolgte eine iterative Weiterentwicklung des Erhebungsinstruments. Die zum Zeitpunkt dieser Veröffentlichung aktuelle Version des Standortfragebogens findet sich im Onlinematerial.

#### Analyse der Umsetzungsverfahren an einzelnen Standorten

Auf Basis der Ergebnisse aus den Umfragen und anderweitiger Rückmeldungen wurden einzelne Standorte gebeten, ihr konkretes Vorgehensmodell zur Einführung und Nutzung der Einwilligungsdokumente in einer der Sitzungen der AG Consent vorzustellen. Ausgewählt wurden insbesondere Standorte mit Besonderheiten in den Antworten, wie etwa sehr hohe Einwilligungszahlen oder stark vom Durchschnitt abweichende Einwilligungsquoten.

#### Analyse der eingesetzten Dokumente

Ab Mai 2022 wurden die Standorte im Rahmen der regelmäßigen Umfrage gebeten, ihre standortspezifisch angepassten und aktuell eingesetzten Einwilligungsdokumente zur Prüfung zur Verfügung zu stellen, sodass eine zentrale Analyse und Bewertung der einzelnen Änderungen in den Dokumenten möglich wurden. Unterschieden wurde dabei zwischen vorgesehenen und laut Handreichung notwendigen Anpassungen, wie etwa der Nennung des Namens der behandelnden und datenschutzrechtlich verantwortlichen Einrichtung und darüber hinausgehenden, in der Handreichung nicht vorgesehenen Änderungen.

### Weiterentwicklung der Dokumente, Materialien und Rahmenbedingungen

Neben dem durch die zuvor beschriebenen Untersuchungsinstrumente generierten Feedback zum Einsatz der Einwilligungsdokumente an den Standorten wurden diverse spontane Anfragen, Rückfragen und Kommentare in der Koordinationsstelle für die AG Consent beantwortet und später systematisch aufbereitet und analysiert. Mit Vertreterinnen und Vertretern von Patientenverbänden wurden zu den Einwilligungsdokumenten sowie zu den Rahmenbedingungen der informierten Einwilligung 2 strukturierte Workshops gemeinsam mit der AG Kommunikation der MII durchgeführt. Zudem wurden am Standort Greifswald Patienten zu ihren Erfahrungen sowie ihrem Verständnis des Einwilligungsprozesses befragt [18]. Weitere Anfragen kamen durch vielfältige Vernetzungsaktivitäten der MII zustande, wie die enge Kooperation mit dem Netzwerk Universitätsmedizin (NUM) und hier insbesondere der Steuerungsgruppe Forschungsinfrastrukturen. Darüber hinaus ist die Koordinationsgruppe Gesundheitsforschungsdateninfrastrukturen zu nennen, in der neben MII und NUM viele weitere Infrastrukturen bzw. Infrastrukturprojekte vertreten sind und die das Ziel hat, synergistisch bei der Infrastrukturentwicklung vorzugehen und insbesondere Doppelentwicklungen zu vermeiden. Ebenso erfolgte ein enger Austausch mit dem Vorstand des Arbeitskreises der medizinischen Ethikkommissionen der Bundesrepublik Deutschland (AK EK).

Alle diese Anfragen und Impulse in Richtung einer Weiterentwicklung der Einwilligungsdokumente, der ergänzenden Materialien und der Rahmenbedingungen für ihren Einsatz wurden in der AG Consent diskutiert und priorisiert. Ein leitender Gedanke war dabei einerseits, dass der bisher gefundene Konsens zu den Einwilligungsdokumenten mit den Datenschutzbehörden und Ethikkommissionen nicht durch Veränderungen der Dokumente infrage gestellt werden sollte und insofern nach Möglichkeit nur modulare Ergänzungen der Dokumente vorgenommen werden sollten. Andererseits sollte durch Aufgreifen neuer Anforderungen und modulare Integration in die Einwilligungsdokumente ein unnötiger paralleler Einsatz multipler Einwilligungsdokumente an den Standorten möglichst vermieden werden.

## Ergebnisse

### Beobachtung und Analyse der Einführung

#### Standortbefragungen

**Abb. 1:**
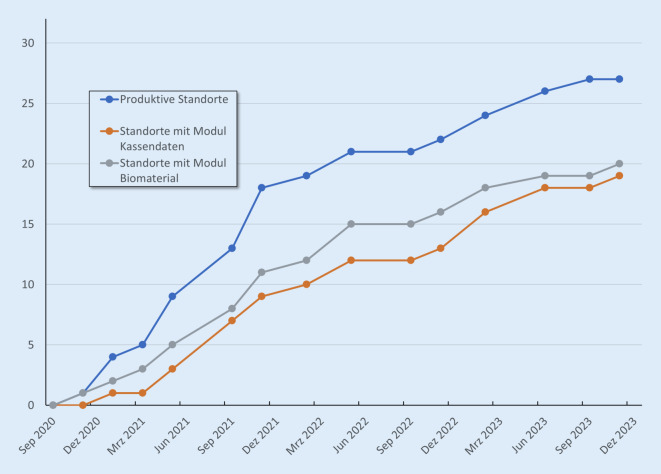
Anzahl der produktiven Standorte im Verlauf. (Quelle: eigene Abbildung)

Im Rahmen der von August 2020 bis November 2023 durchgeführten 15 Umfragen antworteten im Schnitt 77 % der zunächst 30 und ab Mai 2023 32 angeschriebenen Standorte. Insgesamt konnten so 353 Datensätze erhoben werden. Im August 2020 gab es noch keinen Standort mit produktivem Einsatz der Einwilligungsdokumente. Im September 2023 dokumentierten 27 Standorte einen produktiven Einsatz (Anzahl eingeholter Einwilligungen > 0). Die Zahl der Standorte mit produktivem Einsatz des Moduls „Kassendaten“ stieg bis November 2023 auf 19, die der Standorte mit dem Modul „Biomaterial“ auf 20 (Abb. 1).

Neben der Notwendigkeit, dass jedes Nutzungsprojekt mit dem Nutzungsantrag auch ein Ethikvotum der zuständigen Ethikkommission vorlegt, sollten die mit den Einwilligungsdokumenten der MII aufgebauten Datensammlungen (Datenintegrationszentren) an den Standorten auch als Gesundheitsdatenplattformen (Health Databases) im Sinne der Deklaration des Weltärztebundes von Taipeh^3^ ein Votum der für sie zuständigen Ethikkommission einholen. Im November 2023 gaben 21 Standorte an, dass eine Zustimmung der lokalen Ethikkommission zur Einführung der Einwilligungsdokumente vorliegt. Weitere Standorte gaben an, dass eine Abstimmung mit der Ethikkommission erfolgt sei, eine formale Zustimmung aber nicht für erforderlich gehalten werde. Diskussionen mit Standortvertretern hierzu seit Beginn der Umfrage ergaben, dass z. T. eine Prüfung durch die Ethikkommission lediglich im Rahmen konkreter Studienvorhaben als umsetzbar angesehen wurde.

**Abb. 2:**
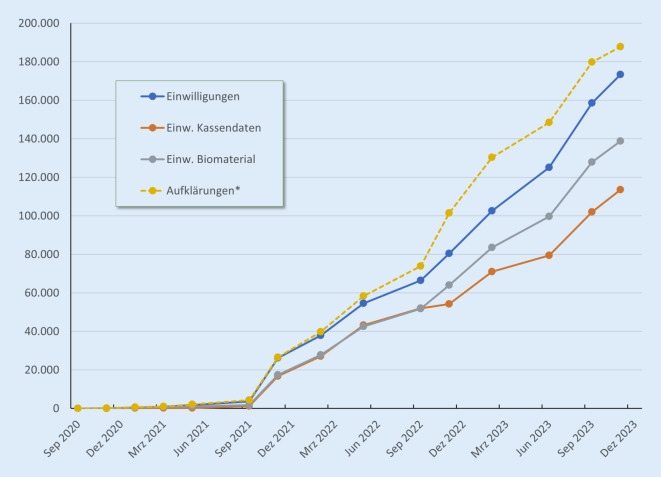
Anzahl der Einwilligungen und Aufklärungen im Verlauf. (*Asterisk* Anzahl der Aufklärungen nicht vollständig erfasst/angegeben). (Quelle: eigene Abbildung)

Die Anzahl der Einwilligungen insgesamt stieg im Umfragezeitraum kontinuierlich auf bis zuletzt 173.289, wobei je nach Zunahme produktiver Standorte zwischen 2 Erhebungszeitpunkten z. T. auch sprunghafte Anstiege zu verzeichnen waren. Die Zunahme pro Monat lag im Jahr 2022 im Mittel bei ca. 5000 Einwilligungen und 2023 bei ca. 7700. Nicht an jeder Umfrage nahmen alle produktiven Standorte teil, sodass für alle hier im Zeitverlauf dargestellten Daten jeweils auf die letzte verfügbare Angabe eines Standorts zum jeweiligen Zeitpunkt zurückgegriffen wurde. Zudem konnten die Angaben im Fragebogen innerhalb eines ca. 14-tägigen Zeitraums eingetragen werden, sodass insgesamt die Zuwachsraten nicht als exakt, sondern als grobe Angaben zur Dynamik im Erhebungszeitraum anzusehen sind. Die Gesamtzahl der Einwilligungen in das Modul „Kassendaten“ betrug bis zum November 2023 113.507, die der Einwilligungen in das Modul „Biomaterial“ 138.802 (Abb. 2).

Die Anzahl der Einwilligungen je Standort war im Erhebungszeitraum sehr unterschiedlich: Von den 27 produktiven Standorten gaben im November 2023 10 Standorte eine gesamte Einwilligungszahl unter 1000 an, 14 eine Zahl zwischen 1000 und 10.000 und 3 Standorte eine Zahl größer als 10.000. Ein Standort allein kam im November 2023 mit einer Zahl von 68.325 auf fast 40 % aller Einwilligungen aller Standorte. Die meisten Standorte gaben zudem an, den Einsatz der Dokumente zunächst auf bestimmte Abteilungen oder Kliniken bzw. z. T. auch auf Pilotprojekte beschränkt zu haben.

Ein Vergleich der Einwilligungsquoten, die beschreiben, wie viel Prozent der Patienten nach erfolgter Aufklärung einwilligen, ist aktuell noch schwierig zu ermitteln. Zum einen wurde die Anzahl der durchgeführten Aufklärungsprozesse nicht von allen Standorten erhoben bzw. im Rahmen der Umfrage angegeben. Die Analyse der Umsetzungsprozesse an einzelnen Standorten hat zudem gezeigt, dass die Angaben zu „erfolgten Aufklärungen“ sehr unterschiedliche Sachverhalte zum Ausdruck bringen. Verlässliche und interpretierbare Ergebnisse zu den Einwilligungsquoten können daher erst dann berichtet werden, wenn ein einheitliches Verständnis und ein Antwortschema hierzu etabliert sind.

Die Dauer der Aufklärung wurde im November 2023 von 19 Standorten mit einem Median von 10 min (Bereich: 1–32 min) angegeben. Betrachtet man nur Standorte mit über 1000 Einwilligungen, kommen 8 Angaben mit einem Median von 7 min (Bereich: 5–13 min) zusammen. Auch diese Angaben sind vor dem Hintergrund eines noch nicht einheitlichen Verständnisses dessen, was eine „erfolgte Aufklärung“ bedeutet, mit Vorsicht zu interpretieren.

#### Analyse der Umsetzungsverfahren

Auf Einladung der AG Consent und z. T. auch eigeninitiativ erfolgten von November 2022 bis März 2023 die Vorstellung und Diskussion von Prozessen und Vorgehensweisen von 6 Standorten in Sitzungen der AG. Schon diese kleine Stichprobe zeigte deutlich heterogene organisatorische Rahmenbedingungen und Vorgehensweisen an den Standorten auf. Die Diskussionen hierzu mündeten dann in Überlegungen, einerseits Mindestanforderungen an den Aufklärungsprozess aus rechtlicher und ethischer Sicht und andererseits begleitende Empfehlungen zur Prozessgestaltung im Sinne einer *Best Practice* an den Standorten zu formulieren. Die Abstimmung dieser Anforderungen und Empfehlungen, die insbesondere auch die unterschiedlichen technischen und organisatorischen Rahmenbedingungen an den Standorten reflektieren müssen, ist noch nicht abgeschlossen.

Parallel zu dieser eher prozessorientierten Untersuchung wurden im Rahmen der Umfrage und z. T. auch separat davon 30 Versionen von Einwilligungsdokumenten, bestehend aus Patienteninformation und Einwilligung, von 26 Standorten für eine Analyse bereitgestellt. Von 5 Standorten liegen nach ersten Rückmeldungen bereits eine zweite Version der Dokumente vor. Der Abgleich dieser eingereichten Dokumente mit den Vorlagen ergab eine Reihe von Abweichungen, die über die Vorgaben der Handreichung zur notwendigen Anpassung der Texte hinausgingen. Diese Abweichungen und ergänzende Anmerkungen wurden individuell an die Standorte zurückgemeldet mit der Bitte um Prüfung und ggf. Anpassung der Texte im Sinne einer weitergehenden Übereinstimmung mit den Vorlagen. Die Sichtung und exemplarische sowie auch überblicksweise Darstellung und Diskussion dieser Abweichungen in der AG hatten zu der Erkenntnis geführt, dass vermutlich an einigen Stellen die bisherige Handreichung zu rigide Vorgaben macht und an anderen Stellen kritische Änderungen an den Texten von den Standorten vorgenommen wurden, die im Sinne der notwendigen Harmonisierung möglichst rückgängig gemacht werden sollten. Aktuell arbeitet ein kleines Team von AG-Mitgliedern an einem Vorschlag zur Klassifizierung dieser Abweichungen, um diese von der AG beschließen zu lassen. Im Ergebnis wird voraussichtlich eine weitere Anpassung der Handreichung, die etwas flexiblere Regelungen enthält, sowie eine erneute Rückmeldung an die Standorte zu den verbliebenen kritischen Änderungen erfolgen. Darüber hinaus wurden Standortanpassungen, die aus Sicht der AG Consent eine Verbesserung darstellen, für die Übernahme in eine zukünftige Version der Standardtexte vorgemerkt.

### Weiterentwicklung der Dokumente, Materialien und Rahmenbedingungen

#### Weitere Dokumente und Versionen

Im Austausch mit den Standorten und weiteren Stakeholdern wurden weitergehende Anforderungen an ein einwilligungsbasiertes Vorgehen zur Bereitstellung von Patientendaten für die Forschung erhoben und in den Sitzungen der AG diskutiert und priorisiert. So entstanden in Ergänzung zu den ursprünglich mit den Datenschutzbehörden und Ethikkommissionen abgestimmten Versionen der Einwilligungsdokumente (Version 1.6d) und der Handreichung (Version 0.9d) weitere Dokumentversionen. An der Erstellung wirkten jeweils Mitglieder der AG und darüber hinaus zahlreiche externe Expertinnen und Experten mit. Alle Dokumente wurden in der AG, z. T. unter Einschluss externer Kompetenz, einem Review unterzogen und dann im Konsensverfahren abgestimmt. Schlussendlich wurden alle Dokumente für die Verwendung in der MII vom NSG freigegeben. Eine Übersicht dieser Dokumentversionen befindet sich in Tab. 1. Alle Dokumente sind zudem auf der Website der MII hinterlegt.^4^

**Tab. 1:** Übersicht weiterer Dokumentversionen*

**Übersetzungen der Ausgangsdokumente**	**Version**
Englische Fassung der Einwilligungsdokumente	1.6d
Englische Fassung der Handreichung	0.9d
Türkische Fassung der Einwilligungsdokumente	1.6d
Arabische Fassungen der Einwilligungsdokumente mit und ohne deutsche Kommentare	1.6d
**Versionen mit inhaltlichen, modularen Ergänzungen**	
Einwilligungsdokumente mit zusätzlichen Modulen mit separaten Einwilligungsoptionen für die Nutzung von Daten aus Voraufenthalten für die Forschung und für den Drittstaatentransfer (Transfer von Daten und ggf. Biomaterial in Länder mit geringerem Datenschutzniveau als in der EU)	1.7.2
Einwilligungsdokumente mit zusätzlichem Modul mit separater Einwilligungsoption für die pandemiebedingte zusätzliche Erhebung und zentrale Nutzung von COVID-19-bezogenen Patientendaten im Rahmen des Projekts CODEX des Netzwerks der Universitätsmedizin	1.6f
Studienmodul Typ I als generischer Text mit separater Einwilligungsoption zum Anhängen an Studieneinwilligungen, um studienspezifische Daten nach Abschluss der jeweiligen Studie nach Maßgabe der Zweckbestimmungen und Regularien der MII für die Forschung breit zur Verfügung zu stellen	0.2
**Versionen für weitere Zielgruppen**	
Einwilligungsdokumente für Kinder im Alter von ca. 7–11 Jahren	1.1
Einwilligungsdokumente für Jugendliche im Alter von ca. 12–17 Jahren	1.1
Einwilligungsdokumente für Eltern/Sorgeberechtigte	1.1
Erläuterung in einfacher Sprache zur Patienteninformation mit und ohne Biomaterialmodul, nach TÜV-zertifiziertem Qualitätsstandard der Firma capito mit dem Gütesiegel „Leicht Lesen“ in der Stufe B1 für Personen mit nichtdeutscher Herkunftssprache, durchschnittlicher Lesekompetenz oder einfacher Bildung	6
**Aktualisierte Handreichung**	
Handreichung mit Berücksichtigung aller ergänzenden Dokumente und Versionen	1.3

* Alle Dokumente verfügbar unter https://www.medizininformatik-initiative.de/de/mustertext-zur-patienteneinwilligung. Zugegriffen: 01.03.2024

Die inhaltlich ergänzenden Module für die Nutzung von Daten aus Voraufenthalten und für den Drittstaatentransfer in der Dokumentenversion 1.7.2 befinden sich derzeit in der Abstimmung mit der Task Force Forschungsdaten der DSK. Diese Abstimmung ist noch nicht abgeschlossen, sodass hier ggf. noch mit Anpassungen zu rechnen ist.

#### Weitere Materialien

Die folgenden zusätzlichen Informationsmaterialien wurden in den letzten 3 Jahren in Ergänzung zu den Einwilligungsdokumenten von der AG erstellt, ebenfalls wieder unter Einbeziehung verschiedener externer Expertinnen und Experten sowie Vertretern der AG Kommunikation der MII:

- Ergänzende Online-Informationen zur Forschung mit genetischen Daten mit Verlinkung in der Patienteninformation der Einwilligungsdokumente (Version 1),^5^
- Informationsvideo zu den Einwilligungsdokumenten in Version 1.6d mit modularen Schnittvarianten für den Ein- oder Ausschluss der Module für Kassendaten oder Bioproben in Deutsch und Englisch,^6^
- Vorlagen für Flyer und Poster zu den Einwilligungsdokumenten, die standortspezifisch angepasst werden können (z. B. mit entsprechenden Kontaktdaten)^7^.

### Weiterentwicklung der Rahmenbedingungen

Die Einführung der Einwilligungsdokumente der MII an den Standorten während der COVID-19-Pandemie hat schnell gezeigt, dass für bestimmte Anwendungsfälle die Notwendigkeit besteht, weitere Ergänzungen in die Einwilligungsdokumente mit aufzunehmen. So wurde im Rahmen des NUM eine zentrale Plattform zur Bereitstellung von Forschungsdaten zu COVID-19 aufgebaut, die COVID-19 Data Exchange Platform (NUM-CODEX; [19]). Der hierfür entwickelte und zu erhebende Standarddatensatz [20] ging nicht wesentlich über das hinaus, was an den meisten Standorten bereits in der Routine erhoben wurde, sodass eine Ergänzung der Einwilligungsdokumente der MII anstelle der Verwendung komplett getrennter Einwilligungsdokumente sinnvoll schien. Diese Einwilligung sollte zusätzlich zu einer je nach Standort etwas über die Routinediagnostik hinausgehenden Datenerhebung auch die Überführung der Daten direkt in eine zentrale Plattform sowie deren Nutzung im Rahmen einer zentral gesteuerten Governance abdecken. Hierfür wurde die Version 1.6f der Einwilligungsdokumente (Tab. 1) entwickelt, die ein entsprechendes Zusatzmodul (Z-Modul) enthält, und innerhalb der MII und des NUM abgestimmt. Derzeit geben in der Umfrage noch 7 Standorte an, dieses ergänzende Modul zu nutzen.

Auch wenn eine breite Nutzung dieses Moduls an den im NUM organisierten Standorten nicht erreicht werden konnte, wurde die Idee intensiv weiterverfolgt, standort- oder auch verbundspezifische Ergänzungen in die Einwilligungsdokumente der MII aufzunehmen. Für solche modularen Ergänzungen, Studienmodul Typ II genannt, wurde ein ausführliches Regelwerk erarbeitet, welches in der aktuellen Handreichung (Tab. 1) im Abschnitt 4.7 beschrieben ist. Derzeit laufen erste Versuche, auf dieser Basis modulare Ergänzungen abzustimmen.

Für die technische Abbildung der verschiedenen Optionen der Einwilligungsdokumente der MII sowie die in einzelnen Antwortoptionen implizit enthaltenen mehrfachen Erlaubnisse (z. B. Nutzung der Patientendaten für 30 Jahre vs. Erhebung weiterer Patientendaten für 5 Jahre) wurden eindeutige *Policies* definiert und deren inhaltliche Definitionen mit der AG abgestimmt [21]. Darauf aufbauend wurde auch ein Modul des Kerndatensatzes der MII für die strukturierte Abbildung der Einwilligungsinformationen entwickelt (siehe auch Beitrag von Ammon et al. in diesem Themenheft). Die Verwaltung und Abfrage dieser strukturierten Informationen können dann in spezialisierter Software erfolgen (z. B. [22]).

## Diskussion

Die hier dargestellte Einführung der Einwilligungsdokumente der MII an den in der MII organisierten universitätsklinischen Standorten zeigt einerseits, dass der Ansatz der Standardisierung von Dokumenten und Rahmenbedingungen und die übergreifende Abstimmung mit Ethikkommissionen, Datenschutzbehörden und weiteren Stakeholdern erfolgreich war. Es ist gelungen, an Standorten mit heterogenen Voraussetzungen identische Formulare einzusetzen und Patientendaten auf dieser Basis nachhaltig und für breit beschriebene Forschungszwecke einheitlich nachnutzbar zu machen, auch wenn die vollständige und dauerhafte Einhaltung der verbindlich abgestimmten Rahmenbedingungen und Standards eine weitere iterative Optimierung durch regelmäßige Analyse- und Rückkopplungsschritte erfordern wird. Die dargestellten Zahlen sprechen dafür, dass so absehbar ein sehr umfangreicher Patientendatensatz für die standortübergreifende Sekundärnutzung zur Verfügung stehen wird.

Andererseits zeigen die hier präsentierten Einwilligungszahlen auch, dass eine Umsetzung an den Standorten in der Fläche über alle Kliniken und Fachabteilungen hinweg noch längst nicht erreicht ist. Die Umsetzung einer solchen Einwilligungslösung stellt die Standorte vor Herausforderungen, für die bislang keine ausreichenden personellen, räumlichen und zeitlichen Ressourcen eingeplant werden können. Um hier zu höheren Einwilligungszahlen und einer damit auch steigenden Repräsentativität der erfassten Patientendaten zu kommen, wird es entscheidend sein, die Prozesse weiter zu optimieren und insbesondere eine möglichst umfassende Vorabinformation der Patientinnen und Patienten über verschickte Unterlagen, Patientenportale sowie die verschiedenen bereitstehenden Informationsmaterialien zu erreichen. Vor diesem Hintergrund arbeitet die AG derzeit mit hoher Priorität an der Ausarbeitung von Minimalstandards zum Aufklärungsprozess und ergänzenden Empfehlungen zur Best-Practice-Gestaltung des Aufklärungs- und Einwilligungsprozesses.

Noch offen, aber von hoher mittelfristiger Relevanz ist die Frage, ob es mit den Einwilligungsdokumenten der MII gelingt, unterschiedliche und parallel genutzte Einwilligungsunterlagen an den Standorten zu ersetzen. Hierfür müssen studien-, standort- und verbundspezifische Erweiterungen so in die Einwilligungsdokumente der MII integriert werden, dass deren verbindlicher und einheitlicher Charakter nicht verloren geht. Erste Versuche, modulare Erweiterungen in diese Dokumente auf Basis des hierzu erstellten Regelwerks zu integrieren, sprechen grundsätzlich für einen erfolgversprechenden Ansatz und die vorsichtige Aussicht auf eine mögliche Reduzierung der Vielfalt von Einwilligungsdokumenten an den deutschen Universitätsklinika, was sowohl eine organisatorische Vereinfachung für die Standorte als auch eine Erhöhung der Transparenz für die Patientinnen und Patienten bedeuten könnte. Dafür ist es entscheidend, das vorhandene Regelwerk für solche Ergänzungen in der Praxis zu testen und bei Bedarf iterativ weiterzuentwickeln.

Trotz der umfangreichen Abstimmung der Einwilligungsdokumente wird in der Literatur stellenweise die Rechtmäßigkeit eines Broad Consent angezweifelt [23], z. T. auch mit direktem Bezug zur Umsetzung in der MII [7, 24]. Dabei ist das Konzept eines Broad Consent (siehe Infobox) in der modernen biomedizinischen Forschung breit anerkannt und wird in vielen Initiativen und Projekten verwendet [12–14, 25–28]. Die MII hat diesen bewährten Ansatz mithilfe besonderer Transparenzinstrumente weiterentwickelt. Die Kritik basiert zentral darauf, dass in Art. 4 Nr. 11 DSGVO eine Einwilligung für den „bestimmten Fall“ gefordert wird, was sich durch eine bloß in einem Erwägungsgrund (Nr. 33 DSGVO) formulierte Öffnung für die Forschung nicht aufweichen lasse. Übersehen wird dabei allerdings, dass die geforderte Festlegung auf den „bestimmten Fall“ der Auslegung bedarf und genau hierfür auch Erwägungsgrund Nr. 33 DSGVO heranzuziehen ist. Auch der Europäische Datenschutzausschuss (EDSA) vertritt in seinen Leitlinien zur Einwilligung diese Sichtweise, auch wenn darauf hingewiesen wird, dass bei besonderen Kategorien personenbezogener Daten, wie z. B. Gesundheitsdaten, strengere Maßstäbe bei der Interpretation des Erwägungsgrunds Nr. 33 DSGVO anzulegen sind [29]. Entsprechend wird in der Literatur auch die Auffassung vertreten, dass ein Broad Consent für die medizinische Forschung unter bestimmten Voraussetzungen zulässig sein kann [30–36]. Zudem wird darauf hingewiesen, dass dem ethischen Prinzip der Informiertheit als Voraussetzung einer Einwilligung auch dadurch entsprochen werden kann, dass explizit über die Offenheit der späteren Verwendung der Daten und ggf. Proben informiert wird [33, 37].

Mit Blick auf alternative Consent-Modelle, wie den Dynamic Consent [38, 39] und den Meta-Consent ([40, 41]; siehe Infobox), wird auch die Erforderlichkeit eines Broad Consent infrage gestellt [7, 24]. Die Umsetzbarkeit solcher Consent-Modelle – zumal potenziell für alle Patienten der deutschen Universitätsklinika – wird dabei kaum hinterfragt, auf Gegenargumente aus der Literatur [42] und der AG Consent^8^ nicht vertieft eingegangen. Letztlich finden sich in der Literatur nur wenige Beschreibungen konkreter Umsetzungen eines Dynamic Consent, so z. B. bei regional begrenzten Projekten [43] oder eingeschränkt auf spezifische seltene Erkrankungen und eine entsprechend überdurchschnittlich interessierte und engagierte Patientengruppe [44]. Übersichtsartikel zum Dynamic Consent gehen entsprechend immer wieder auf dieselben wenigen Beispiele ein [39, 45, 46]. Auch die Leitlinien des EDSA zur Einwilligung schlagen einen Dynamic Consent beispielhaft als Lösungsmöglichkeit vor, wenn die Zwecke bei der Datenerhebung noch nicht so spezifisch angegeben werden können. Letztlich wird ein Dynamic Consent hier aber nicht vorgeschrieben [29].

Und auch zum Meta-Consent, bei dem die Betroffenen zu Beginn festlegen können, nach welchem Einwilligungsmodell sie beteiligt werden möchten, gibt es in der Literatur Hinweise darauf, dass Betroffene wenig Interesse an einer dauerhaft feingranularen Steuerung von multiplen Einwilligungen haben oder diese aus technischen Gründen gar nicht wahrnehmen können [47, 39, S. 652]. Für onkologische Patienten gibt es zudem empirische Hinweise darauf, dass ein Broad Consent gegenüber einem Dynamic Consent favorisiert wird, insbesondere wenn dadurch der Nutzen für die Forschung erhöht wird [48].

Letztlich zeigt die hier dargestellte breite Beteiligung der Standorte, dass sich die umfassende Abstimmung dieses Einwilligungsmodells mit den Datenschutzbehörden und Ethikkommissionen gerade auch vor dem Hintergrund der weiterlaufenden juristischen Fachdebatte bewährt hat.

Die Erfahrungen mit dem Aufbau einer zentralen Forschungsplattform für Daten aus der Versorgung der Patienten mit COVID-19 [19] haben einerseits gezeigt und bestätigt, dass auch während einer Pandemie die bestehenden gesetzlichen Rahmenbedingungen nicht für eine einheitliche einwilligungsfreie Bereitstellung der Daten genutzt werden konnten (vgl. hierzu [2]). Andererseits hat sich gezeigt, dass zu spezifische Einwilligungen in solchen Zeiten problematisch sind und gerade unvorhergesehene Forschungsnotwendigkeiten nicht unterstützen können. Zudem gelingt gerade in Zeiten eines bereits extrem belasteten Gesundheitssystems nicht die Einführung neuer, aufwändiger Einwilligungsprozesse, die, wie die hier dargestellten Erfahrungen zeigen, doch erhebliche Ressourcen benötigen. Insofern besteht die Hoffnung, dass eine weitere Nutzung des hier vorgestellten Broad Consent, der zudem nicht bei jedem neuen Behandlungsfall von den Patientinnen und Patienten eine erneute Einwilligung erfordert, auch für kommende Pandemien eine bessere Datenlage mit unterstützen kann. Gleichzeitig sind auch die hier offenbar gewordenen Defizite im Bereich einwilligungsfreier Forschung in der Debatte zur Ausgestaltung der rechtlichen Rahmenbedingungen in Deutschland angesprochen worden.

### Der Broad Consent im Kontext sich wandelnder gesetzlicher Rahmenbedingungen

Mit dem Data Governance Act, dem Data Act und insbesondere dem European Health Data Space (EHDS) stehen mehrere Regelungsansätze zur Verbesserung der Rahmenbedingungen für die Datennutzung in der medizinischen Forschung auf europäischer Ebene vor der Tür. Im nationalen Rahmen wird dies – trotz der beschränkten Gesetzgebungskompetenz auf Bundesebene – durch gleich mehrere Gesetzesvorhaben der Bundesregierung ergänzt und vorbereitet. Neben den geplanten Forschungsdaten‑, Medizinforschungs- und Registergesetzen ist hierbei insbesondere das Gesetz zur Nutzung von Gesundheitsdaten zu gemeinwohlorientierten Forschungszwecken und zur datenbasierten Weiterentwicklung des Gesundheitswesens (Gesundheitsdatennutzungsgesetz – GDNG) zu nennen, welches im Dezember 2023 im Bundestag abschließend diskutiert und abgestimmt wurde und im Februar 2024 auch den Bundesrat passiert hat.

Für datenverarbeitende Gesundheitseinrichtungen sieht das Gesetz in § 6 Abs. 3 bestimmte Befugnisse für die einwilligungsfreie Sekundärnutzung von Behandlungsdaten u. a. zu Forschungs- und Qualitätssicherungszwecken vor. Dazu gehört auch die Möglichkeit des Teilens der Behandlungsdaten im Rahmen von öffentlich geförderten Zusammenschlüssen von datenverarbeitenden Gesundheitseinrichtungen, einschließlich Verbundforschungsvorhaben und Forschungspraxen-Netzwerken, wenn die Interessen der verantwortlichen Einrichtungen die Interessen der betroffenen Patientinnen und Patienten erheblich überwiegen und die zuständige Datenschutzaufsichtsbehörde zustimmt. Neben einer Strafbewehrung bei missbräuchlichem Umgang mit den Daten sind auch Transparenzpflichten zur Datennutzung, wie sie auch der Broad Consent der MII vorsieht, Bestandteil der gesetzlichen Vorgaben des GDNG. Das Zusammenwirken mit weiteren Gesetzen, wie z. B. den bestehenden Regelungen im Datenschutz- und Krankenhausrecht auf Landesebene, einschließlich der ebenfalls aktuell in Verhandlung befindlichen europäischen EHDS-Verordnung, muss sich im Detail noch zeigen, ebenso müssen Erfahrungen zum Abstimmungsprozess mit den Datenschutzbehörden zu einzelnen Vorhaben des Datenteilens gemacht werden. Unabhängig hiervon wird der Broad Consent insbesondere eine elementare Voraussetzung bleiben für (a) das Teilen von Daten mit Einrichtungen jenseits der im Gesetz genannten gemeinsamen Nutzung durch öffentlich geförderte Zusammenschlüsse datenverarbeitender Gesundheitseinrichtungen, was wohl auch Kooperationen mit der Industrie und internationalen Partnern betrifft; (b) das Einlagern, Verarbeiten und Teilen von Proben zu Forschungszwecken; (c) die ergänzende Nutzung von Datenquellen, die nicht unter das GDNG fallen, wie etwa den bei den Krankenkassen verfügbaren Daten, einschließlich erforderlicher Datenverknüpfungen, sowie (d) die Nutzung der in der Einwilligungserklärung der MII geregelten Rekontaktierungsoptionen.

Bei einer Weiterentwicklung der gesetzlichen Rahmenbedingungen hin zu einer gesetzlich geregelten Datenspende [6] könnten die hier dargestellten Einwilligungsverfahren und -dokumente auch als Modell für ein gesetzlich normiertes *Opt-in* dienen. In jedem Fall bleiben die mit dem Einwilligungsprozess etablierten Mitwirkungsmöglichkeiten und Transparenzangebote wichtig, um die gesellschaftliche Akzeptanz der Datennutzung zu erhalten und auszubauen. Die optimale Nutzung der Chancen durch die sich dynamisch weiterentwickelnde Rechtslage wird eine wichtige zukünftige Aufgabe der MII, ihrer AG Consent und ihrer Partner sein.

#### Infobox Broad, Dynamic und Meta-Consent^9^

*Broad Consent*: Die breite Einwilligung ermöglicht es Patientinnen und Patienten, der Verwendung ihrer Daten und ggf. Proben ohne Einschränkung auf ein bestimmtes Projekt oder eine bestimmte Fragestellung zuzustimmen. Festgelegt ist hingegen ein bestimmter Forschungsbereich, dem sich spätere Daten- und ggf. Probennutzungen nachvollziehbar zuordnen lassen müssen. Die breite Einwilligung trägt der Tatsache Rechnung, dass gerade bei modernen, datengetriebenen Forschungsansätzen häufig nicht vorhergesagt werden kann, welche Daten in welcher Form für zukünftige Fragestellungen relevant sind. Durch die größere Offenheit bei der späteren Daten- und ggf. Probennutzung bestehen besondere Anforderungen an den Schutz der Daten und ggf. der Proben sowie die Kontrolle und Transparenz in Bezug auf deren spätere Nutzung.

*Dynamic Consent*: Bei einem dynamischen Einwilligungsprozess werden moderne Informationstechnologien genutzt, um Patientinnen und Patienten auf Basis entsprechender Kommunikation interaktiv in die Nutzung ihrer Daten und Proben für unterschiedliche Forschungsprojekte einwilligen zu lassen. Dies ist grundsätzlich gerade auch für generische Forschungsinfrastrukturen wie die MII relevant. Es bestehen aber Herausforderungen in Bezug auf die konkrete Umsetzung und die konkreten Auswirkungen dynamischer Einwilligungsprozesse, beispielsweise auf die Repräsentativität von Daten oder eine planbare Nutzung von Daten für wissenschaftlich valide Auswertungen; diese potenziellen Bias-Effekte sind bisher noch nicht vollständig untersucht.

*Meta-Consent*: Der Ansatz der Meta-Einwilligung bringt unterschiedliche grundlegende Einwilligungsformen zusammen. Er soll es Patientinnen und Patienten ermöglichen, für verschiedene Arten künftiger Forschungsprojekte unterschiedliche Präferenzen bzgl. der Einwilligung zu definieren. Die Optionen reichen von einer pauschalen, breiten Einwilligung oder Ablehnung bis hin zur Präferenz der dynamischen Einbeziehung. In der Praxis ist dieses Konzept bislang kaum erprobt.

### Supplementary Information

In der Online-Umfrage genutzter Fragebogen

## Footnotes

1 Siehe https://www.datenschutzkonferenz-online.de/media/pm/20200427_Einwilligungsdokumente_der_Medizininformatik-Initiative.pdf. Zugegriffen: 01.03.2024.

2 https://soscisurvey.de. Zugegriffen: 01.03.2024.

3 WMA-Declaration of Taipei on Ethical Considerations regarding Health Databases and Biobanks (Oktober 2016).

4 https://www.medizininformatik-initiative.de/de/mustertext-zur-patienteneinwilligung. Zugegriffen: 01.03.2024.

5 Abrufbar unter www.vernetzen-forschen-heilen.de/genetische-daten; zum Zeitpunkt des Erscheinens dieses Artikels wird voraussichtlich schon eine deutlich erweiterte Version 2 abrufbar sein. Zugegriffen: 01.03.2024.

6 Eine vollständige Version in Deutsch ist unter https://www.medizininformatik-initiative.de/de/mustertext-zur-patienteneinwilligung einsehbar, weitere Versionen können auf Anfrage zur Verfügung gestellt werden. Zugegriffen: 01.03.2024.

7 Werden auf Anfrage mit den standortspezifischen Daten in Druckauflösung erstellt und zur Verfügung gestellt.

8 Stellungnahme der AG Consent der Medizininformatik-Initiative zu patientenindividueller Datennutzungstransparenz und Dynamic Consent unter https://www.medizininformatik-initiative.de/de/mustertext-zur-patienteneinwilligung. Zugegriffen: 01.03.2024.

9 Eine ausführliche Auseinandersetzung der AG Consent der MII mit den verschiedenen Consent-Optionen findet sich unter https://www.medizininformatik-initiative.de/sites/default/files/2019-09/MII_AG-Consent_Stellungnahme-Consent-Modelle_v05.pdf. Zugegriffen: 01.03.2024.
