# Supplementary material for: Nationally standardized broad consent in practice: initial experiences, current developments, and critical assessment
Source: Bundesgesundheitsblatt Gesundheitsforschung Gesundheitsschutz. 2024 Apr 19;67(6):637–47. [Article in German] doi: 10.1007/s00103-024-03878-6 (PMC11166792; doi:10.1007/s00103-024-03878-6)
Supplement: Supplementary file 1 — In der Online-Umfrage genutzter Fragebogen [file 103_2024_3878_MOESM1_ESM.pdf]

Seite 01

Die Arbeitsgruppe (AG) Consent der Medizininformatik-Initiative des BMBF (MII) hat Muster-Einwilligungstexte erarbeitet, bestehend aus einer Patienteninformation und einer Einwilligungserklärung. Ergänzend wurde eine Handreichung für den Einsatz an den Standorten erstellt, die über die notwendigen bzw. empfohlenen Rahmenbedingungen für den Einsatz der Einwilligungsdokumente informiert.

Die Einwilligungsdokumente sollen an den Standorten in den Konsortien der MII einführend eingesetzt werden. Um festzustellen, wie der Einwilligungsprozess in den Klinikalltag eingegliedert werden kann, führt die AG Consent konsortienübergreifend eine Evaluierung des Einwilligungsprozesses durch. Im Vordergrund steht dabei die Verbesserung und Optimierung des Einwilligungsprozesses, ggf. auch durch eine Anpassung der Dokumente.

Achtung: Füllen Sie diesen Fragebogen nur mit Bezug zu den Einwilligungsdokumenten der AG Consent der MII und deren sachgerechter Anpassung (siehe zugehörige Handreichung) aus. Der Fragebogen dient nicht dazu, den Einsatz anderweitiger Einwilligungsdokumente an den Standorten zu untersuchen.

Dieser Fragebogen dient nicht der Erhebung personenbezogener Daten. Nachverfolgungseinstellungen (z.B. Cookies) wurden daher deaktiviert. Bitte achten Sie auch darauf, lediglich standortbezogene und keine auf einzelne Personen beziehbare Angaben in den Fragebogen einzutragen.

Bitte nehmen Sie sich etwas Zeit (ca. 10 min), um die Fragen für Ihren Standort zu beantworten. Die AG Consent dankt Ihnen für Ihre Unterstützung.

1. Welchen MII-Standort vertreten Sie?

Bitte wählen Sie Ihren Standort aus (Achtung: Dies ist die einzige verpflichtende Angabe des Fragebogens, ohne eine Auswahl kommen Sie nicht auf die nächste Seite.):

Standort: [Bitte auswählen]

2. Wie wurde die Entscheidung zur Nutzung der Einwilligungsdokumente der MII an Ihrem Standort getroffen?

Mehrfachauswahl möglich

- [ ] Der **Vorstand** war in die Entscheidung eingebunden.
- [ ] Die **Klinikleitung** war in die Entscheidung eingebunden.
- [ ] **Sonstige Gremien** waren in die Entscheidung eingebunden: __________

3. Liegen Zustimmungen des lokalen Datenschutzbeauftragten und der Ethikkommission zur Nutzung der Einwilligungsdokumente der MII vor?

	(noch) nicht angefragt	angefragt	Zustimmung liegt vor	Ablehnung liegt vor	Sonstiges (siehe Erläuterung)
a) Zustimmung des lokalen Datenschutzbeauftragten:	○	○	○	○	○
b) Zustimmung der lokalen Ethikkommission:	○	○	○	○	○

Erläuterung: __________

4. Werden die Einwilligungsdokumente an Ihrem Standort allen Patienten vorgelegt?

- ○ ja
- ○ nein

5. Wenn nein, was sind die Gründe und Kriterien für die Einschränkung?

a) Was sind die Gründe für die Einschränkung? __________

b) Nach welchen Kriterien wird die Auswahl der Patienten entschieden (z.B. Art der Erkrankung, Fachabteilung, Alter)? __________

c) Auf welche Abteilungen/Kliniken ist die Anwendung der Einwilligungsdokumente beschränkt? __________

6. Welches Informationsmaterial der MII wird an Ihrem Standort im Rahmen der Aufklärung der Patienten genutzt?

Mehrfachauswahl möglich

- [ ] Flyer der MII für Patienten
- [ ] Film zur Einwilligung mit Ton
- [ ] Film zur Einwilligung ohne Ton, mit Untertiteln
- [ ] Poster
- [ ] Sonstiges: ____________________

7. Wie werden die Informationsmaterialien an Ihrem Standort genutzt?

Mehrfachauswahl möglich

- [ ] Die Flyer liegen bei uns öffentlich aus.
- [ ] Die Flyer werden digital präsentiert (z.B. auf Tablets).
- [ ] Die Flyer sind bei uns online zugreifbar.
- [ ] Der Film zur Einwilligung wird im Aufnahmebereich gezeigt.
- [ ] Der Film wird nur ausgewählten Patienten gezeigt.
- [ ] Der Film ist auf der Website abrufbar.
- [ ] Das Poster hängt im Aufnahmebereich.
- [ ] Andere Nutzung der Materialien: ____________________

8. In welcher räumlichen Situation findet der Aufklärungs- und Einwilligungsprozess an Ihrem Standort im Regelfall statt?

Mehrfachauswahl möglich

- ☐ in der Aufnahme
- ☐ im Patientenzimmer
- ☐ im Behandlungszimmer
- ☐ auf dem Flur
- ☐ in speziell dafür reservierten Räumen
- ☐ in sonstigen Räumen: ____________

9. Welche Hilfsmittel werden an Ihrem Standort für die Prozessunterstützung bzw. das Qualitätsmanagement genutzt?

Mehrfachauswahl möglich

- ☐ Es gibt eine **SOP** (Standard Operating Procedure) zur Nutzung der Einwilligungsdokumente der MII.
- ☐ Es gibt eine **Checkliste** zur Nutzung der Einwilligungsdokumente der MII.
- ☐ Es gibt **sonstige Hilfsmittel** zur Nutzung der Einwilligungsdokumente der MII. ____________

10. Welche Berufsgruppen führen an Ihrem Standort im Regelfall das Aufklärungsgespräch durch?

Mehrfachauswahl möglich

- ☐ Ärzte
- ☐ Pflegepersonal
- ☐ Aufnahmepersonal
- ☐ spezifisches Personal für den Aufklärungs- und Einwilligungsprozess
- ☐ sonstiges Personal: ____________

11. Wird das an Ihrem Standort für den Aufklärungsprozess eingesetzte Personal hierfür geschult?

- ○ Ja, in dieser Form: ____________
- ○ Nein

12. Zu welchem Zeitpunkt wird in der Regel die Aufklärung zur Einwilligung durchgeführt?

- ○ Mit der Aufnahme in das Krankenhaus
- ○ Im weiteren Verlauf der Behandlung
- ○ Nach der Behandlung
- ○ Zu einem anderen Zeitpunkt: ____________

13. Wie lange dauert im Durchschnitt das Aufklärungsgespräch?

____ Minuten

14. In welcher Form wird die Einwilligung an Ihrem Standort in der Regel eingeholt?

- a) Die Einwilligung wird in Papierform eingeholt ○
- b) Die Einwilligung wird digital eingeholt ○
- c) Sowohl als auch (z.B. in unterschiedlichen Abteilungen) ○
- Wenn a) oder c): Das unterschriebene Formular wird eingescannt ☐
  - Wenn ja: Das Papierformular wird nach dem Scannen vernichtet (ersetzendes Scannen) ☐

15. In welchem elektronischen Erfassungssystem werden die strukturierten Einwilligungsinformationen an Ihrem Standort in der Regel für die weitere Nutzung erfasst?

Mehrfachauswahl möglich

- ☐ Krankenhausinformationssystem (KIS)
- ☐ generic Informed Consent Service (gICS)
- ☐ Thieme eConsent pro
- ☐ Sonstige Software: ____________

16. Welche der optionalen Module der Einwilligungsdokumente der MII werden an Ihrem Standort genutzt?

Mehrfachauswahl möglich

- [ ] Kassendaten (Versionen 1.6d, 1.6f, 1.7.2)
- [ ] Biomaterial (Versionen 1.6d, 1.6f, 1.7.2)
- [ ] Biomaterial mit Option für Add-On-Samples (Versionen 1.6d, 1.6f, 1.7.2)
- [ ] Retrospektive Daten (Version 1.7.2)
- [ ] Drittstaatentransfer (Version 1.7.2)
- [ ] Zusatzmodul für NUM-CODEX (Version 1.6f)

17. Welche der folgenden ergänzenden Versionen der Einwilligungsdokumente oder Erläuterungen zu den Dokumenten setzen Sie an Ihrem Standort ein?

Mehrfachantwort möglich

- [ ] Version in englischer Sprache (Basis ist Version 1.6d, Link)
- [ ] Version in türkischer Sprache (Basis ist Version 1.6d, Link)
- [ ] Version in arabischer Sprache (Basis ist Version 1.6d, Link)
- [ ] Erläuterung zur Patienteninformation in einfacher Sprache (Basis ist Version 1.6d, Link)
- [ ] Version für Kinder (7-11 Jahre, Basis ist Version 1.7.2, Link)
- [ ] Version für Jugendliche (12-17 Jahre, Basis ist Version 1.7.2, Link)
- [ ] Version für Eltern/Sorgeberechtigte (Basis ist Version 1.7.2, Link)
- [ ] Ergänzung von Studieneinwilligungen (Studienmodul Typ I, Basis ist Version 1.6d, Link)

18. Von Ihrem Standort liegen in der Koordinierungsstelle der MII leider noch keine auswertbaren Einwilligungsdokumente vor. Für die Weiterentwicklung der Einwilligungsdokumente benötigt die AG Consent jedoch einen Einblick in die tatsächliche Verwendung der Vorlagen an den Standorten. Laden Sie hierzu bitte die von ihnen konkret im Einsatz befindlichen Dokumente im Word- (.doc oder .docx) oder PDF-Format (*.pdf) hier hoch.

Sie können bis zu vier Dateien hier hochladen.

1. Datei: Durchsuchen... Keine Datei ausgewählt.
2. Datei: Durchsuchen... Keine Datei ausgewählt.
3. Datei: Durchsuchen... Keine Datei ausgewählt.
4. Datei: Durchsuchen... Keine Datei ausgewählt.

19. Wann wurde an Ihrem Standort die erste Aufklärung einer Patientin oder eines Patienten zu den Einwilligungsdokumenten der MII vorgenommen?

Datum der ersten Aufklärung: TT.MM.JJJJ

20. Bitte geben Sie uns aktuelle Zahlen zu den folgenden Fragen an (sofern Sie diese erfassen), immer gezählt ab dem Zeitpunkt der ersten Aufklärung zu den Einwilligungsdokumenten der MII.

Bitte geben Sie auch eine 0 ein, wenn passend. Lassen Sie das Antwortfeld hingegen leer, wenn die Bedingung für die Frage auf Ihren Standort nicht zutrifft.

a) Wie viele Aufklärungsprozesse wurden an Ihrem Standort durchgeführt? Bitte nur angeben, wenn tatsächlich alle Aufklärungen unabhängig davon, ob Patienten auch eingewilligt haben oder nicht, auch erfasst und gezählt werden.

b) Wie viele Patienten haben in die Datennutzung eingewilligt?

c) Wenn das Kassendaten-Modul genutzt wird, wie viele Patienten haben in diese Option eingewilligt?

d) Wenn das Biomaterial-Modul eingesetzt wird, wie viele Patienten haben in diese Option eingewilligt?

e) Wenn mit dem Biomaterial-Modul auch die Option für Add-On-Samples genutzt wird, wie viele Patienten haben in diese Option eingewilligt?

f) Wie viele stationäre Patienten haben bislang eingewilligt?

g) Wie viele ambulante Patienten haben bislang eingewilligt?

21. Wie viele Widerrufe der Einwilligungen sind an Ihrem Standort seit der ersten Aufklärung zu den Einwilligungsdokumenten der MII eingegangen? Bitte geben Sie hier nur die Zahl tatsächlich erfolgter Widerrufe von vorher bereits getätigten Einwilligungen an. Kein Widerruf in diesem Sinne ist es z.B., wenn eine Patientin direkt nach der Aufklärung die Einwilligung ablehnt.

Bitte geben Sie auch eine 0 ein, wenn passend.

Zahl der Widerrufe: [ ]

22. Ermöglichen Sie abgestufte Widerrufe der Einwilligungen (z.B. nur bezogen auf ein Modul oder eine Option der Einwilligung)?

- ○ Ja
- ○ Nein

23. Wenn ja, geben Sie bitte die jeweilige Anzahl von Widerrufen seit der ersten Aufklärung zu den Einwilligungsdokumenten der MII an Ihrem Standort an.

Bitte geben Sie auch eine 0 ein, wenn passend. Lassen Sie das Antwortfeld hingegen leer, wenn die Bedingung für die Frage auf Ihren Standort nicht zutrifft.

a) Wenn das **Kassenmodul** genutzt wird, Widerrufe insgesamt für das Kassenmodul: [ ]

b) Wenn das **Kassenmodul** genutzt wird, Widerrufe für die Nutzung der Daten **aus den letzten 5 Jahren**: [ ]

c) Wenn das **Kassenmodul** genutzt wird, Widerrufe für die Nutzung der Daten **aus den kommenden 5 Jahren**: [ ]

d) Wenn das **Biomaterialmodul** genutzt wird, Widerrufe für das Biomaterialmodul: [ ]

e) Widerrufe für die **Rekontaktierungsoption für Nacherhebungen**: [ ]

f) Widerrufe für die **Rekontaktierungsoption für die Informierung über Zusatzbefunde**: [ ]

24. Wer nimmt an Ihrem Standort Widerrufe der Patienten entgegen?

Stelle für Widerrufe: [ ]

25. Wer nimmt an Ihrem Standort sonstige Anfragen zu Betroffenenrechten (z.B. Auskunft, Berichtigung) entgegen?

Stelle für sonstige Betroffenenrechte: [ ]

Vielen Dank für die Beantwortung aller Fragen!

Achtung: Mit einem Klick auf "Beenden" wird der Fragebogen abgeschlossen. Der Abschluss sollte erst erfolgen, wenn wirklich alle Fragen beantwortet wurden.

Vielen Dank für Ihre Teilnahme!

Wir möchten uns ganz herzlich für Ihre Mithilfe bedanken.

Ihre Antworten wurden gespeichert, Sie können das Browser-Fenster nun schließen.
